# Editorial: Self-assembled biodegradable materials for medical sensing, diagnosis, and therapy

**DOI:** 10.3389/fchem.2023.1174976

**Published:** 2023-03-08

**Authors:** Kuan Hu, Hongyu Meng, Zhou Li

**Affiliations:** ^1^ National Institutes for Quantum and Radiological Science and Technology, Chiba, Japan; ^2^ Beijing Institute of Nanoenergy and Nanosystems, Chinese Academy of Sciences (CAS), Beijing, China

**Keywords:** self-assemble, biodegadable, sensing, diagnosis, therapy

As the COVID-19 pandemic has passed, the suffering it caused will not be forgotten quickly. It is essential that humanity comprehends the significance of biomedical technology in fighting major contagious illnesses. Self-assembled lipid nanoparticles have been particularly instrumental in combating the mortality rate caused by COVID-19 infections. Since the successful implementation of self-assembled nanomaterials in cancer nanomedicines, its potential has been further explored in more far-reaching areas. It is believed that biodegradable nanomaterials, which are self-assembled, can provide a promising approach to dealing with various diseases, particularly incurable cancers and newly discovered infectious diseases. Taking advantage of the ordered arrangement of molecular monomers, self-assembled materials possess many desirable physical and biological characteristics, making them suitable for medical sensing, diagnosis of illnesses, and treatment.

The recent success of vaccines has highlighted the importance of exploring the potential of self-assembled materials. To ensure the swift arrival of the next industrial revolution, it is essential to be aware of the current state of the field and the potential for future progress. This research topic has recently published 6 papers, including two original research articles and four review articles, which cover a range of topics from peptides and polymers to hydrogels, and their applications in the biomedical field.

Recently, sonodynamic therapy has been viewed as a potential treatment for breast cancer. Nevertheless, the internal GSH-mediated redox system inside cancer cells may compromise its effectiveness. In an effort to address this problem, Luo et al. designed a self-assembled IR@CPGel that can target glucose-6-phosphate dehydrogenase (G6PD) to examine redox homeostasis and enhance the oxidative stress microenvironment of tumour tissues, thereby improving the effectiveness of sonodynamic therapy for breast cancer. The hydrogel composite is highly biocompatible and low in toxicity, making the self-assembled IR@CPGel a plausible locally injectable drug for clinical trials.

Despite efforts, the synthesis of cost-effective, biocompatible and safe self-assembled nanomaterials for biomedical purposes remains a challenge. Fluorescent carbon dots (CDs) have been demonstrated to be useful in various areas such as sensing, bioimaging, and photoelectronic devices. With the aim of producing solid CDs in a cost-effective and straightforward manner, Luo et al. presented a methodology for the direct production of solid CDs from collagen waste, achieving a remarkable conversion yield and a hierarchically structured CDs. Furthermore, the prepared CDs demonstrated outstanding Fe^3+^ sensing capacity and great biocompatibility.

In addition to the two original research articles, four review articles exploring magnetic hydrogels, self-assembling peptide coatings, peptide-based immunotherapy materials, and self-assembled/composited lignin colloids have been released, offering a broad overview of the rising and extensive uses of self-assembled biodegradable materials in various areas of the biomedical field. Magnetic hydrogels with ordered structures and anisotropic properties exhibit tremendous advantages, enabling them to have broad applications in drug delivery, soft robotics, biosensing, and tissue engineering. Sun et al. reviewed the synthetic methods for magnetic hydrogels, followed by a summary of their functionalities and biomedical applications. The challenges and prospective outlook of magnetically ordered hydrogels are also given. Peptides serve as a pivotal material source for self-assembling materials owing to their remarkable biocompatibility, biodegradability, and versatile functionalities. Two published reviews focused on the biomedical applications of self-assembled peptide materials. Tian et al. described in their review the recent advances in self-assembling peptide matrices as functional coatings for implantable devices. Their work implied the potential clinical benefits of peptide surface engineering in improving the safety issues of implantable devices. Wang et al. paid attention to the emerging innovative cancer treatment modality cancer immunotherapy, introducing the applications of self-assembled peptide materials in cancer vaccines, immunodrug carriers, and adjuvants, as well as clinical investigations of these materials. Given the great clinical success of immunotherapy, it is anticipated that peptide-based immunotherapy nanomaterials may hold a very bright future in combating various diseases in the future. Except for peptides, lignin, a naturally renewable source, is attracting intense attention in biomedical fields. Zhou et al. described the assembly behavior of lignin in different solvents, introduced various methods for preparing lignin composite colloids, and highlighted their applications in therapy, cosmetics, and emulsification.

In recent years, researchers have been striving to overcome the challenges that impede the clinical use of self-assembling biodegradable materials. These challenges include the need for homogeneous and reproducible fabrication, as well as a greater understanding of the biological effects of these materials. This topic provides an overview of the latest developments and advancements in the field in order to inspire innovative ideas, promote interdisciplinary research, and ultimately facilitate the transition of self-assembling materials from the laboratory to the clinic.

